# Brain theory

**DOI:** 10.3389/fncom.2014.00114

**Published:** 2014-10-16

**Authors:** Dmitri Krioukov

**Affiliations:** Department of Physics, Northeastern UniversityBoston, MA, USA

**Keywords:** complex systems, complex networks, Bayesian brittleness, modeling and simulations, theoretical physics

Inspired by the BRAIN initiative, a quickly growing body of brightest minds worldwide are set to understand how the brain works (Alivisatos et al., 2012). How soon should we expect this goal to be achieved? Is this goal achievable at all? And how wide is the consensus on what the goal really is? It is difficult to expect any agreement on an answer, if there is no agreement on the question.

The interpretation of the question varies dramatically from one research group to another and even within a single group. Quite naturally, everyone seems to have his/her own opinion about what such a broad and elusive problem as “how the brain works” might mean. At one extreme are simplicists who do not see any problem at all. At the other extreme are metaphysicists dealing with the problem of consciousness, and how to “explain the feeling of the red color” to a person blind at birth. Somewhere in between, perhaps closer to the former group, are those who believe that complete brain mapping at the neuron level will solve the problem. But there seems to be no argument that the problem of brain mapping at the neuron level will not solve the problem of explaining the red to a blind. Having the brain map handy may be a necessary but definitely not sufficient condition for understanding *how the brain works*. Knowing the structure is not enough. We also have to know the dynamics, and understand the laws that govern it.

This state of things is not unique for understanding the brain. It is fairly typical for any dynamical system. The brain is somewhat atypical in that this dynamical system is very complex. And it is very atypical in that this complex system is being studied by itself. Here I avoid discussing the latter aspect any further, and comment on the former.

It is difficult to name a field of science that made no contribution to studying complex dynamical systems. Biologists, physicists, mathematicians, computer scientists—all bring their own methods, knowledge, and intuition to advance our understanding of complex biological systems such as the brain. Yet compared to how physics advances at the Large Hadron Collider, for example, our understanding of biological system appears to advance more slowly and erratically. Why? The answer that may very well be correct is that the brain is more complex than the Higgs boson. But this answer misses the point.

There is an impressive gap in how modern physics approaches “simple” and “complex” systems. For the “simplest systems,” by which I mean the fundamental interactions in nature, we now have a simple fundamental theory (Ryder, 1996). It starts with figuring out the group of symmetries of a given system. Then some (usually the simplest) scalar invariant, called action, of this group is identified. The least action principle is finally applied to this action, resulting in the so called Euler-Lagrange equations that fully specify the laws of the system dynamics. This theory describes not only all the fundamental interactions in nature—the electromagnetic, weak, and strong interactions, and gravity in general relativity—but also many less fundamental phenomena, such as classical mechanics (Landau and Lifshitz, 1976). In that case the group of symmetry consists of rotations in space, and translations in space or time, while the Euler-Lagrange equations are Newton's laws.

There exists no even remote analogy of this theory for complex systems. In our increasingly computerized times, we energetically collect increasing volumes of data—BIG DATA—about many complex systems including the brain. Bigger data, coupled with smaller but swifter computers, strongly suggest physicists to give up and turn into computer scientists. It seems indisputable that data mining, machine learning, and other data-driven approaches are superior to any theoretical investigations if one is to obtain quick answers about big data. It often gets overlooked that these answers sometimes do not answer any questions, or that the same publication first formulates a question that did not exist before, and then answers it. One can easily find lines of research or even publication venues in which, given a collection of challenge datasets, the algorithms endlessly compete in accuracy of predicting the known. Successful attempts to predict or learn something useful that we do not already know about the data are rare but stellar, because such predictions turn out to be very challenging, and there are many reasons why.

Perhaps the most obvious and frequently mentioned reason is that complex systems are very stochastic or even chaotic—these features are often perceived synonymous to “complex.” Even an infinitesimally small mistake in modeling the system can grow exponentially large. Studying complex systems in computer science or statistics often involves modeling. Many models have many parameters, and there exists an impressive body of literature in statistics about the dangers associated with such subjects as model selection or data overfitting (Attias, 2000; Burnham and Anderson, 2002). Roughly, any model with a sufficiently large number of parameters, applied to any data, can predict anything one wants to predict—hence the ease of predicting the known. Yet until recently many people believed George Box who said that “all models are wrong, but some are useful” (Box and Draper, 1987). Unfortunately the second half of this statement is wrong according to the recent results on *Bayesian brittleness* in statistics (Owhadi et al., 2013a,b). The essence of these results is that if model *X* generates some data, but we use model *Y* to study it, then the results of our studies can grow arbitrarily wrong, even if our model *Y* is arbitrarily close to model *X*, quite contrary to the common belief that a close enough model must be good enough. From the physics perspective, these results seem to suggest that the “phase space” of models is a phase space of a chaotic system. Does this chaos mean that we should completely abandon our attempts to understand how the brain works?

Let us consider a simple deterministic chaotic system, such as a double pendulum or three gravitating bodies for example, and imagine a simple mortal who does not know much physics or mathematics, but who has full access to big observational data on these systems. Will s/he be able to quickly understand the very simple laws that fully determine the dynamics of these systems appearing so complex? The task seems next to impossible! By all means, the brain is more complex than a double pendulum, and the scientists studying it may be not so simple mortals, buy why do many of us believe that there are no simple laws describing this apparent complexity, if the whole history of science is a neverending demonstration of the point above—of how blind we are at reverse engineering of even simple physical systems?

The history of gravity and astronomy is another classic example of this blindness (Dreyer, 1953). This history, with Aristotle, Ptolemy, Copernicus, Galilei, Newton, and Einstein as key brightest minds, took some twenty four centuries to unfold. The critical episode of moving the center of the universe from the Earth (Ptolemy) to the Sun (Copernicus) took just fifteen centuries. There are some reasons to believe (but no preserved evidence) that Hypatia could be close to Copernicus'es discoveries, but her atrocious execution—likely ordered by Cyril, the Bishop of Alexandria, who got later canonized—ended her quest (Dzielska, 1996), and we had to wait another 1000 years. Today we do have a simple and elegant theory of gravity, which falls into the universal least-action theory (Wald, 2010), but this simplicity and elegance are *post-factum*. They are results of centuries of data collection, struggle, uncertainty, and maddening mistakes.

Coming back to the brain, it may be the case that there is no simple universal theory describing how the brain and other complex systems work, but we do not have any indications for that to be true. The history of science so far indicates the opposite. Many systems and phenomena that we initially perceived as very complex and intractable, later turned out to be described by rather simple fundamental laws or their interactions. We were just blind, and could not see them right away. There are also more specific, rather than historical, indications that such laws might exist for complex systems that can be represented as networks, of which the brain is a paradigmatic example. These indication include certain structural and dynamical universalities observed across many complex networks (Boccaletti et al., 2006), as well as emerging evidence that the same least-action canonical theory may describe their dynamics (Krioukov et al., 2012).

This way or the other, one thing is certain: as far as the brain and other complex systems and networks are concerned, we are at a rather Ptolemaic stage, collecting the data, awaiting for Copernicus. Forget about Einstein for a time being.

## Conflict of interest statement

The author declares that the research was conducted in the absence of any commercial or financial relationships that could be construed as a potential conflict of interest.
